# Supplementary material for: Genome-wide identification, characterization and gene expression of BES1 transcription factor family in grapevine (Vitis vinifera L.)
Source: Sci Rep. 2023 Jan 5;13:240. doi: 10.1038/s41598-022-24407-y (PMC9816167; doi:10.1038/s41598-022-24407-y)
Supplement: Supplementary file 3 — Supplementary Information. [file 41598_2022_24407_MOESM3_ESM.zip › Vvi_Atr/Vitis_vinifera.PN40024.v4.dna_sm.toplevel.fa.vs.Amborella_trichopoda.AMTR1.0.dna_sm.toplevel.fa.html/Atr-AmTr_v1.0_scaffold00174.html]

|  |  |  |  |  |  |  |  |  |  |  |  |  |  |
| --- | --- | --- | --- | --- | --- | --- | --- | --- | --- | --- | --- | --- | --- |
| Duplication depth | Reference chromosome | Collinear blocks | | | | | | | | | | | |
| 0 | Atr-ERN14465 |  |  |  |  |  |  |  |  |  |  |  |  |
| 0 | Atr-ERN14466 |  |  |  |  |  |  |  |  |  |  |  |  |
| 0 | Atr-ERN14467 |  |  |  |  |  |  |  |  |  |  |  |  |
| 0 | Atr-ERN14468 |  |  |  |  |  |  |  |  |  |  |  |  |
| 0 | Atr-ERN14469 |  |  |  |  |  |  |  |  |  |  |  |  |
| 0 | Atr-ERN14470 |  |  |  |  |  |  |  |  |  |  |  |  |
| 0 | Atr-ERN14471 |  |  |  |  |  |  |  |  |  |  |  |  |
| 0 | Atr-ERN14472 |  |  |  |  |  |  |  |  |  |  |  |  |
| 0 | Atr-ERN14473 |  |  |  |  |  |  |  |  |  |  |  |  |
| 0 | Atr-ERN14474 |  |  |  |  |  |  |  |  |  |  |  |  |
| 0 | Atr-ERN14475 |  |  |  |  |  |  |  |  |  |  |  |  |
| 0 | Atr-ERN14476 |  |  |  |  |  |  |  |  |  |  |  |  |
| 0 | Atr-ERN14477 |  |  |  |  |  |  |  |  |  |  |  |  |
| 0 | Atr-ERN14478 |  |  |  |  |  |  |  |  |  |  |  |  |
| 0 | Atr-ERN14479 |  |  |  |  |  |  |  |  |  |  |  |  |
| 0 | Atr-ERN14480 |  |  |  |  |  |  |  |  |  |  |  |  |
| 0 | Atr-ERN14481 |  |  |  |  |  |  |  |  |  |  |  |  |
| 0 | Atr-ERN14482 |  |  |  |  |  |  |  |  |  |  |  |  |
| 0 | Atr-ERN14483 |  |  |  |  |  |  |  |  |  |  |  |  |
| 0 | Atr-ERN14484 |  |  |  |  |  |  |  |  |  |  |  |  |
| 0 | Atr-ERN14485 |  |  |  |  |  |  |  |  |  |  |  |  |
| 0 | Atr-ERN14486 |  |  |  |  |  |  |  |  |  |  |  |  |
| 0 | Atr-ERN14487 |  |  |  |  |  |  |  |  |  |  |  |  |
| 0 | Atr-ERN14488 |  |  |  |  |  |  |  |  |  |  |  |  |
| 0 | Atr-ERN14489 |  |  |  |  |  |  |  |  |  |  |  |  |
| 0 | Atr-ERN14490 |  |  |  |  |  |  |  |  |  |  |  |  |
| 0 | Atr-ERN14491 |  |  |  |  |  |  |  |  |  |  |  |  |
| 0 | Atr-ERN14492 |  |  |  |  |  |  |  |  |  |  |  |  |
| 0 | Atr-ERN14493 |  |  |  |  |  |  |  |  |  |  |  |  |
| 0 | Atr-ERN14494 |  |  |  |  |  |  |  |  |  |  |  |  |
| 0 | Atr-ERN14495 |  |  |  |  |  |  |  |  |  |  |  |  |
| 0 | Atr-ERN14496 |  |  |  |  |  |  |  |  |  |  |  |  |
| 0 | Atr-ERN14497 |  |  |  |  |  |  |  |  |  |  |  |  |
| 0 | Atr-ERN14498 |  |  |  |  |  |  |  |  |  |  |  |  |
| 0 | Atr-ERN14499 |  |  |  |  |  |  |  |  |  |  |  |  |
| 0 | Atr-ERN14500 |  |  |  |  |  |  |  |  |  |  |  |  |
